# Supplementary material for: Association between oral microbiome and seven types of cancers in East Asian population: a two-sample Mendelian randomization analysis
Source: Front Mol Biosci. 2023 Nov 21;10:1327893. doi: 10.3389/fmolb.2023.1327893 (PMC10702768; doi:10.3389/fmolb.2023.1327893)
Supplement: Supplementary file 1 [file DataSheet1.ZIP › Supplementary Materials/MR plots for tongue/tongue═╝/Pancreatic cancer/╥╚╧┘░⌐_╔╕╤í╜ß╣√/pheno.236_to_pancreatic cancer_forest.pdf]

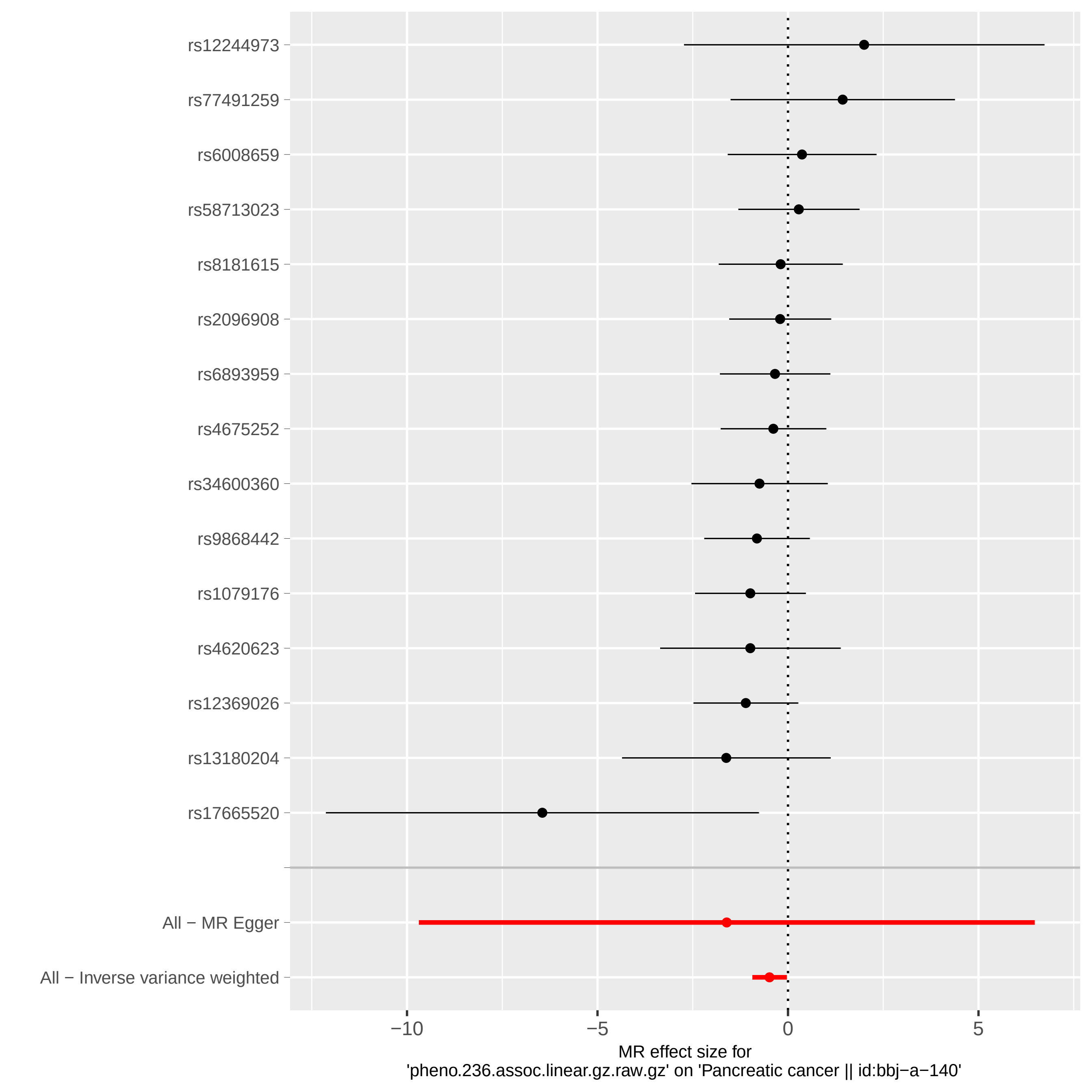
rs12244973
rs77491259
rs6008659
rs58713023
rs8181615
rs2096908
rs6893959
rs4675252
rs34600360
rs9868442
rs1079176
rs4620623
rs12369026
rs13180204
rs17665520
All − MR Egger
All − Inverse variance weighted
−10
−5
0
5
MR effect size for
'pheno.236.assoc.linear.gz.raw.gz' on 'Pancreatic cancer || id:bbj−a−140'
